# Concussions in Sledding Sports and the Unrecognized “Sled Head”: A Systematic Review

**DOI:** 10.3389/fneur.2018.00772

**Published:** 2018-09-18

**Authors:** Melissa D. McCradden, Michael D. Cusimano

**Affiliations:** ^1^Department of Neurosurgery, St. Michael's Hospital, Injury Prevention Research Office, Toronto, ON, Canada; ^2^Dalla Lana School of Public Health, University of Toronto, Toronto, ON, Canada

**Keywords:** sport-related concussion, subconcussion, bobsleigh, luge, skeleton, injury prevention, sled head, sledding sports

## Abstract

**Background:** Sport-related concussion is a significant public health concern. Little research has been conducted on sport-related concussion and injury prevention strategies in competitive sledding sports like bobsleigh, luge, and skeleton. Athletes have identified “sled head” as a key concern due to its symptom burden.

**Purpose:** To summarize our knowledge of the prevalence of concussion and related symptoms in sledding sports; to utilize Haddon's Matrix to inform and define strategies for injury prevention.

**Methods:** An independent information specialist conducted a search for the known literature on injuries in non-recreational sledding sports, and specifically for concussion via OVID Medline, CINAHL, the Cochrane Database, EMBASE, PsycInfo, PubMed, Scopus, and the Web of Sciences from 1946 to December 2017. After iterative searches of reference sections, a total of 844 articles were assessed for inclusion.

**Results:** Nine articles were included for review. Concussions are a common occurrence in elite sledding sport athletes, affecting 13-18% of all sledding athletes. Significant variance exists between events, indicating a potential effect of the ice track in injury risk. The condition known as “sled head” is discussed and identified as a key point of further investigation. A number of potential injury prevention strategies are discussed.

**Interpretation:** Head injuries and concussions are an important injury for elite sledding sports and a number of avenues exist for prevention. More work is required to delineate the mechanisms, characteristics, natural history and management of “sled head.”

## Introduction

Concussion is a significant public health concern particularly for sport and recreation-related mechanisms of injury (1, 2). Sledding (tobogganing) is a common recreational activity for which traumatic brain injuries (TBIs) represent 9% of all sledding injuries (3), though individual trauma centers have reported up to 37% (4, 5). An injury to the head is the leading cause of serious injury sustained in snow sport accidents in children (6), and is a significant risk factor for death following sledding accidents in both adults and children (7). The severity of head injuries can be mitigated with the use of helmets (5, 8, 9), but there is continued resistance among winter sport participants to their routine use (10). While other winter sports such as, ice hockey, skiing, snowboarding, and tobogganing have received ample attention in concussion research, sledding sports have received relatively little.

Competitive sledding—also known as “sliding sports” and includes luge, skeleton, and bobsleigh (Table 1)—has received surprisingly little attention in concussion research. As a result, we know fairly little about the incidence of concussion. The danger is certainly present, as sledders mount their apparatuses and slide as fast as possible down an icy, curving track. While recreational toboggans can reach around 40 km per hour (11), sledding athletes more than double these speeds. Even at the Olympics, winter sports carry the potential for devastating brain injuries. The tragic fatal crash of Georgian luger Nodar Komaritashvili during the 2010 Olympic Games drew a critical eye to safety issues in the sport (12).

**Table 1:** Characteristics and comparison of sledding sports.

	**Bobsleigh**	**Luge**	**Skeleton**
Rider(s) position	Sitting	Lying face-up, feet-first	Lying face-down, head-first
Events	Monobob, 2-person, 4-person (men only)	Single and double rider	Single rider
Initiation of movement	15 m running start	Grabs handles on track to propel self forward	Running start
Steering mechanism	Pulley system	Handles, leg muscle pressure on runners, shifting body weight	Shifting head, shoulders, and body weight, drag toe
Braking	Yes	No–use of leg muscles	No–drag feet
Speed	Over 150 km/hr	Over 100 km/hr	Over 100 km/hr

*Provides descriptive and biomechanistic qualities of bobsleigh, luge, and skeleton (sledding sports)*.

Also concerning are anecdotal reports of “sled head,” described as headaches, fogginess and occasionally with dysequilibrium resulting mostly from bumpy or multiple track runs (13–15). Though not described in the literature this problem is serious enough that it is well-known among sledding athletes, and these concerns are significant enough to prompt some skeleton and luge athletes to withdraw from competitions where the ice track is “too bumpy,” limit training runs prior to competitions, and prompted the Head Coach of the 2014 Sochi Olympic skeleton team to institute a maximum number of training runs down the track in the lead-up to the Games (15). Sledders called for more awareness of brain injuries in sledding sports (16), and have openly described the challenges of concussion and sled head in the media (17, 18). Whether sled head is a concussion, distinct from concussion, represents a form of subconcussion, or due to neck strain is unclear.

Identification and documentation of concussions is essential to strategic reduction of concussion incidence to improve the safety of sledding for athletes. To describe the current state of knowledge about concussion in sledding, we conducted a systematic literature review of all sledding competitive sports: luge, skeleton, and bobsleigh. As these sledding sports are virtually exclusively competitive due to their speeds and specialization, we limit our search to include sledding athletes (i.e., excluding tobogganing). Our research question was defined as “what is the prevalence of concussion and concussion-like symptoms in competitive sledding athletes?”

## Methods

A systematic review of the literature was conducted on 1 May 2018 for all articles published from 1946 to April 2018: OVID Medline, CINAHL, the Cochrane Database, EMBASE, PsycInfo, PubMed, Scopus, and the Web of Sciences were all searched to identify citations relevant to sledding sports and concussion for the purpose of conducting a scoping review. Inclusion criteria of primary research papers were: (1) involved Olympic-sanctioned sledding sports (i.e., bobsleigh, luge, skeleton); (2) quantification of concussion or other injuries, as well as illnesses. Exclusion criteria were: (1) alcohol or drug intoxication at the time of the incident concussion; (2) moderate or severe head injury; (3) recreational population. In reference to investigations of potential sled head, we included “illness” as an inclusion criteria and specifically scanned articles for mention to any concussion-like symptoms that may potentially be related to sled head (as it is described by athletes). No strict age range was observed in the review, though it is noted that there are very few youth (< 18) sledding athletes and almost all competitive sledders are over 16 years of age.

**Figure 1:**
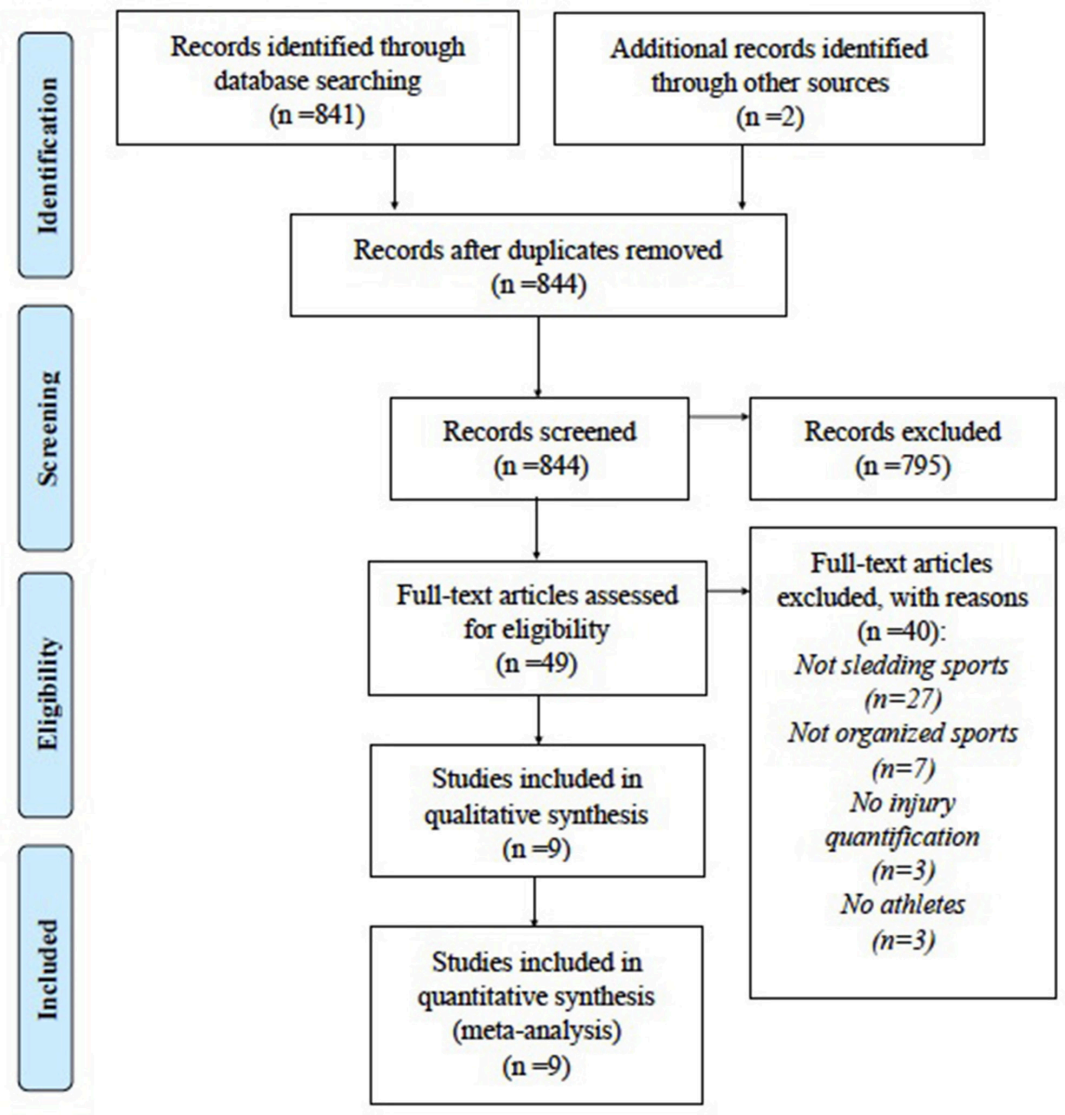
PRISMA Diagram of article abstraction. From Moher et al. (23).

An information specialist working independently submitted 841 documents for review, after duplicates were removed. Two additional citations were identified through searching of reference sections of included articles. Forty-nine articles were assessed for eligibility based on our criteria. From these, 40 were excluded on the basis of: not involving a sledding sport population (*n* = 27), not involving an organized sport group (i.e., competitive; *n* = 7), no documentation of injuries (*n* = 3), not involving athlete subjects (*n* = 3). We were left with nine articles which were included in the final analysis (Figure 1). The majority of studies presented are the result of the International Olympic Committee's (IOC) injury surveillance system (ISS) to collect standardized quantification of musculoskeletal injuries, concussions, and illnesses (24), as other sources were limited.

## Results

### Concussions/head injuries in sledding sports

In the Salt Lake City 2002 Winter Olympics (25), sledding athletes were among the most frequent patients presenting to affiliated injury clinics, accounting for 19.6% of all patients (bobsled, 10.1%; luge, 8.7%; skeleton, 0.7%). A similarly high utilization of emergency services by sledding sport athletes was observed in the 2006 Torino Olympic Games (26).

At the 2010 Olympics (19), 7% of all registered athletes reported concussions, which was twice as high as the concussion incidence in the 2008 Summer Olympic Games (24). Engbretsen et al. (19) report 30 head injuries accounting for 10.5% of all injuries, and 20 concussions were recorded. Of these, only two resulted in the athlete being absent at least 1 week from their sport; for eight concussions the length of absence was missing, and there were 10 concussions from which athletes returned to sport in less than a week. Six head injuries and five concussions were sustained by sledding sport athletes.

At the 2014 Sochi Olympics (21), 11 concussions occurred, representing an overall rate of 0.4%, which was half the rate observed in Engebretsen et al. (19). Injuries from sledding sports represented 14% of all injuries. In the 2016 Lillehammer Youth Olympic Games (22), two concussions and one head injury were sustained by sledding sport athletes, representing a concussion incidence of 1.4%, but again these were not specified within sledding events. Similarly, of the eight sledding injuries at the 2012 Youth Olympic Games (20), two were head injuries and one was a concussion, again without to reference to the specific sledding event. There is extremely little information about the mechanisms of injury incidence, other than that they most often occur during training (19–21).

A safety audit of the Vancouver Olympic track in Whistler, BC was performed by analyzing video footage of runs that resulted in incidents and injuries (12). Risk of injury was highest at the lower curve sections, and for runs starting from higher, and risk dropped significantly after a participant had exceeded 150 runs. Only 63% of incidents were documented in full in incident reports, indicating there is a substantial amount of missing information. The authors also acknowledge that the severity of many injuries was not determined. Their injury rate was similar to that of a safety audit conducted at the Lake Placid Olympic training site in New York (25). Full results of concussion incidences and their breakdown by specific sport are presented in Table 2.

**Table 2:** Representation of concussion among sledding sport athletes.

**Event and reference**	**Sport**	**Athletes**	**Injuries**	**Concussion (% of all injuries)**
2010 Olympic Games (19)	Bobsleigh Luge Skeleton *All sledding* ***All sports***	159 47 159 *365* ***2,567***	20 6 2 *28* ***287***	5 (25%) 0 0 *5 (18%)* ***20 (7%)***
2012 Youth Olympic Games (20)	Bobsleigh Luge Skeleton *All sledding* ***All sports***	36 70 28 *134* ***1,015***	3 4 1 *8* ***111***	? ? ? *1 (13%)* ***8 (7%)***
2014 Olympic Games (21)	Bobsleigh Luge Skeleton *All sledding* ***All sports***	171 108 47 *326* ***2,788***	31 9 5 *45* ***391***	? ? ? ? ***11 (3%)***
2016 Youth Olympic Games (22)	Bobsleigh Luge Skeleton *All sledding* ***All sports***	29 70 40 *139* *1,131*	4 2 7 *13* ***108***	? ? ? *2 (15%)* ***12 (11%)***

*Depicting the studies from the literature review that identified the prevalence of concussion in individual sledding events (where available), for all sledding sports (italicized), and for all winter sports assessed in the study (bold)*.

### Luge

Luge athletes in general had fewer injuries than skeleton and bobsleigh athletes. Seven percent of all luge athletes required treatment at a medical clinic during the 2006 Torino Games (26). At the 2010 Vancouver Winter Olympic Games (19), luge was among the sports with the lowest injury incidence overall with 2% of all luge athletes sustaining injuries, and none reported a concussion. One injury had death as an outcome. At the 2012 Youth Olympic Games (20), 6% of all luge athletes were injured. In Sochi (21), 8.3% of all luge athletes were injured, which more often occurred during competition than in training. The Lillehammer Youth Olympic Games (22) recorded 2.9% of luge athletes sustaining injuries.

Cummings et al. (27) abstracted the records from 1,043 luge athletes (24% female) between 12 and 35 years of age who attended a sport medicine clinic and were training at the USA Olympic Training Center in Salt Lake City, New York. Of all injuries necessitating removal from training for at least 2 days, head injuries represented 20% of all injuries, of which 47% were diagnosed as concussion. Concussion therefore represented 2.5% of all luge injuries. Sixty-four percent of injuries were sustained in crashes, indicating the athlete bumped the edge of the track with or without loss of control of the luge, was thrown from the sled, or failed to stop the sled at the base of the track and collided with the barrier.

### Bobsleigh

Engebretsen et al. (19) noted that bobsleigh was among the sports with the highest percentage of the team sustaining an injury (20% of all bobsledders), and was on par with ice hockey, freestyle skiing, snowboard cross, and short track speed skating in this regard. Five bobsledders sustained a concussion, representing 25% of the concussed athletes. Male and females bobsledders were equally likely to sustain injuries. Others have similarly reported that bobsled athletes have a high percentage of utilization of medical services (25, 26), and consistently have one of the highest rates of injury (19, 22). In Sochi, 18.1% of bobsledders were injured (21), and 1.2% of these injuries necessitated more than 7 days reprieve from sport. In the Lillehammer Youth Olympic Games, 13.8% of bobsledders were injured (22).

### Skeleton

Engebretsen et al. (19) reported only three injuries in two skeleton athletes (one male, one female) representing 6% of the whole team, and no injuries were concussions. Similarly, the 2002 Olympics saw a small representation of skeleton athletes among clinic patients (25), although it is not reported what percentage of all skeleton athletes was injured. Piat et al. (26) found at the Torino Olympics that skeleton athletes had the largest utilization (26%) among the three sports (bobsleigh, 17%; luge 7%), and of all winter sports had the highest percentage of athletes requiring treatment. Similarly in Sochi (21), 10.6% of the skeleton team was injured, most of which were injured during training and none required more than a day's reprieve from sport. The Lillehammer Youth Olympic Games (22) reported that 17.5% of all skeleton athletes were injured. To the contrary, in the 2012 Youth Olympic Games (20), only one skeleton athlete was injured (4% of all skeleton athletes).

### “sled head”

Despite the review process and further exhaustive manual searching of the literature, we identified only one narrative report from 1986 discussing the viewpoint of one physician who describes how common headaches are among luge athletes (14). Roos describes eight luge athletes who reported post-run headaches, which were noted to be “almost universal” among lugers. Some lasted for minutes, others, for days; as the number of runs increased, so did the likelihood and severity of headaches. They presented bilaterally, were described as “throbbing” or “constant,” and progressed in severity throughout a day of training, limiting the number of runs an athlete could perform. No other details of coinciding symptoms such as, dizziness or light sensitivity are described, or whether they are routinely assessed.

## Discussion

For bobsleigh, luge, and skeleton, concussions account for 13–15% of all injuries to sledding sport athletes at high-level competitions [i.e., the Olympics; (19–22)], yet little is known about how concussions occur due to the paucity of research conducted thus far. This is surprising given the surge in popularity in sledding sports in the past 20 years, with a combined nine events and more teams qualifying for the Olympics each year. It is therefore difficult not only to draw conclusions about the prevalence of concussion in sledding sports, but also prevention efforts. This is particularly concerning given that sledding sports appear to have among the highest overall injury incidences of Olympic winter sports (19, 21, 22, 25, 26), though with considerable variability between studies. To put the problem into context, the studies included in this review found that concussions from hockey represented 3–19% of all hockey injuries; in ski and snowboard, concussion represented 3–11% of all injuries; in sledding sports, concussion represented between 13 and 25% of injuries (19, 21, 22). These data indicate that attention to concussion injuries in sledding athletes is an important piece of overall injury management.

While concussions sustained in crashes are the most obvious mechanism of injury, reports of “sled head” are plentiful in between athletes. Concerns were substantial enough to prompt 2014 Olympic Team Canada head coach Duff Gibson to implement a policy allowing a maximum of three runs per day for skeleton athletes (15). His choice was informed not by scientific research, but by anecdotal evidence that sled head increases with more runs, especially on bumpy track. An early report from 1986 appears to corroborate the evidence that bumpy track is associated with headaches (14). Whether the headaches these athletes report following runs down the track represent concussion, subconcussive head impacts, or muscle strain is currently unknown. However, the multitude of athletes reporting concussion symptoms in association with heavy run volumes is extremely concerning, given that there are no concrete guidelines for sledding athletes and management of sled head. Bobsleigh Canada Skeleton (BCS) has implemented a concussion policy that disallows athletes with concussion symptoms from training until they are symptom-free (28). Further research is needed to ensure that athletes are not incurring repeated brain damage as a result of concussive and subconcussive forces leading to debilitating symptoms.

Given that the literature on the mechanisms of concussion in sledding sports is so limited, we draw from academic studies of general injury patterns, sport organization policies, and media-reported events to inform recommendations for prevention of sledding-related concussions.

### Avenues for the prevention of head injuries

We utilized Haddon's Matrix (29) as a framework for exploring potential host, agent, and environment factors (Table 3).

**Table 3:** Haddon's Matrix of concussion prevention in sledding sports.

	**Host**	**Agent**	**Environment**
Pre-event	Track experience Visualization strategies Strength/technique Concussion education	Improved shock absorption Energy absorption layer Retractable shield (bobsled)	Presence of medical staff Compliance with track safety standards Ice condition standardized
Event	Technique Mouthguard and helmet use Mental state	Prompt retrieval of sled	Maintenance of ice conditions
Post-event	Recognize, facilitate and encourage reporting of concussion	Inspection	Injury documentation Regular performance and documentation of safety audits

*Presents potential injury prevention measures as a function of the host, agent, and environment for pre-event, during the event, and post-event time periods*.

#### Analysis of host factors

The “host” in our case refers to a sledding sport athlete. Host factors prior to, during, and following the event can contribute to injury risk.

##### Pre-event host factors

Familiarity with the track is associated with lower injury risk (12), as it likely minimizes errors which can result in injury. Athletes employ visualization techniques such as, “track runs” (physically walking down the track and strategizing their movements) to supplement with physical runs to improve their ability to maneuver on the track. Reinforcement of strength and technique is also believed to lessen the risk of injury. Neck strength is felt to be particularly crucial for luge and skeleton athletes, who must hold their head stable while incurring substantial forces up to 5*g*s on curves (30). In 2- and 4-person bobsledding and 2-person luge, athletes describe teamwork and chemistry as essential factors to a smooth drive, particularly for coordinating the start.

Attention to sport-related concussions has resulted in sporting organization adopting concussion policies and mandatory training sessions for coaches. BCS's concussion policy (28) requires that coaches complete the “Concussion Awareness” program provided by the Coaching Association of Canada but it is not clear whether and how this is verified and enforced by BCS. There are no current guidelines for management of sled head.

Ability to react in the moment is believed to be crucial for success in skeleton (31), which would suggest that pre-run simulations might help substantially in reducing head injury. Enhancing meditative, reflective and concentration strategies aimed at calming athletes' minds and to focus on technique and pre-run visualizations to minimize errors may also be an avenue for intervention. However, pre-race performance is not always predictive of performance in competition (32).

##### Event-related host factors

The use of helmets is essential to minimize severe head and brain injury in the event of a high speed crash. Further work on sledding-specific helmets and their characteristics to protect against concussion is required. Given the repetitive impacts at a high speed and transmission of forces from the face and jaw to the brain, use of a mouthguard may also help confer some protection against concussive injury (33), and against symptoms such as, headache (34).

In the event of a skeleton or luge crash, athletes must make certain decisions to try and minimize injury, such as, whether or not to let go of the sled. Training and review of prior visualized crashes to determine high risk events may help in this regard.

##### Post-event host factors

Education of athletes, coaches, trainers and other local personel to recognize and approapriately manage concussion is essential to prevent further concussion in symptomatic athletes. Attention to potential concussion symptoms, prompt medical attention, and awareness of proper rehabilitation protocol can reduce the effects of concussion. Concussion education programs are essential to improving concussion management, but this knowledge must be continually reinforced (35). Symptoms pertaining to mental health are not well-recognized among athletes, coaches, and medical personnel (36). Attention to these and other symptoms following not just crashes but multiple runs down the track will ensure athletes get the care they need to recover.

### Analysis of agent factors

The “agent” refers to the sleds used by sledding athletes; luge and skeleton sleds differ greatly from bobsleds by weight, construction, and safety considerations.

#### Pre-event agent factors

Two specific adjustments to skeleton and luge sleds may potentially reduce concussion symptoms typical of sled head. The first would be to add shock absorbers to the bridge and runners to reduce the bouncing effect on the rider when ice quality is sub-par. The second would be to introduce an energy absorption layer similar to what has been adopted in helmet technology for contact sports. Helmets with pneumatic padding are effective at reducing forces applied to the skull (37, 38). If sleds added a similar energy absorption layer between the saddle and the rider, it could potentially reduce certain symptoms (e.g., headaches).

For bobsleigh, it may be worth considering the addition of a retractable plexiglass shield to protect the heads of the riders in the event that the sled tips over. Currently, when the sled tips over and continues down the track the athletes' heads come into aggressive contact with the sides of the track. A retractable shield would prevent this sort of mechanism of injury, though would not entirely prevent the problem of the head bumping into a hard surface after tipping over.

#### Event-related agent factors

In a crash, athletes typically try to stay inside the bobsled, while skeleton and luge athletes have to decide whether or not to abandon their sled or try to rescue the run. Several uphill sections exist throughout the track, and so sleds that are slowing down can reverse direction and travel backward, which could further injure an athlete laying on the track.

#### Post-event agent factors

Inspection of sled for any damage is important for ensuring the sled remains in good condition according to international regulations (39, 40). The FIL does not have stringent guidelines for helmet safety standards (39), while standards for skeleton and bobsleigh are slightly more strict 40, but are subject to each athlete's national federation.

### Analysis of environment factors

In our analysis of sledding sports, the “environment” includes the track, barriers, and surrounding environment and the social environment.

#### Pre-event environment factors

A race physician with emergency care knowledge and a paramedic must be present at all sanctioned events (39, 40). The track must be maintained to a high standard, and race technicians are charged with removing or minimizing any identifiable risk factors for injury (39, 40). The condition of the ice ought to be standardized and verified for compliance with safety standards at all events. It has been anecdotally noted that sub-par track conditions prompted several athletes to withdraw from World Cup-level events due to concerns of sled head (13). When there is a delay in treatment of the track athletes can lose valuable training time, which can also reduce injury risk, as noted above.

#### Event-related environment factors

During the final training and competitive events, no changes may be made to the ice other than repairing damaged areas, and FIL specifies that “the ice must be superbly prepared.” When a crash happens, track officials are charged with ensuring the track is free and clear of debris, and attending to the dents in the ice caused by edges of runners and sleds scraping the track.

#### Post-event environment factors

Medical personnel must be able to quickly access, attend to and remove an athlete from the track to prevent further injury. Standards for the attendance of medical personnel at elite level sledding events are specified in international regulations (39, 40). Medical personnel should be versed in all aspects of trauma management and access to rapid evacuation to local trauma centers would minimize time to treatment, particularly in the event of more severe injury.

Injury documentation and safety auditing are important for reviewing any incidents where track conditions may have contributed to injury. Following the Vancouver Olympics recommendations from the FIL and IBSF were incorporated into the design of the Sochi track, which was constructed with additional uphill segments that have the effect of slowing down the sled. The Pyeongchang track was developed under the guidance of the International Olympic Committee with the input from bobsleigh, luge, and skeleton federations. This sort of enforcement and oversight is essential to preventing injury and promoting a culture of safety in sledding sports. Proper documentation in full is important to identifying key points in the track where safety improvements can be made, and transparency of these adjustments can improve athletes' and coaches' trust in the safety of a track. Given the discrepancies across Olympic Games of the representation of the individual sledding sports in health service utilization and injury rates (25, 26), it is possible that the individual track elements (i.e., difficulty, speed) contribute substantially to the risk of injury at a given event.

### Culture shock

As has been noted among skiers and snowboarders (10), safety measures must be integrated into the culture of a sport in order to truly make it safer. The greatest challenge for injury prevention in sledding sports is that modifications that could make sleds safer and potentially reduce the risk of concussion also have the potential to slow them down and “change the sport.” The addition of shock absorbers and an energy absorption layer can potentially increase the surface area which could increase drag to slow down the sled. At first pass, no athlete, no coach and no sporting organization will want to make that sacrifice — unless it is adopted by all competitors. Therefore, actions mandated by the IBSF and FIL that clearly define mandatory safety standards for sleds, tracks and athletes must be a priority. Prioritization of brain health must be incorporated into the mandates of sporting organizations in order to change the culture of sport to be more conducive to proper treatment of concussion.

An important improvement could be made in how we record and document injuries at elite sports competitions like the Olympics. The accepted method of quantifying number of injuries by body part (41) with a separate quantification of concussion leaves open the question of whether there were head injuries that were not concussion, or whether not all concussions were coded as head injuries. We herein suggest that all head injuries be classified according to standard clinical criteria such as, those by the Center for Disease Control, the International Classification of Diseases–10th edition or another recognized format.

### Limitations

The inclusion criteria may have introduced a selection bias for studies which relied on medical personnel to report injuries and excluded single case reports and commentaries. In high stakes events such as, the Olympic Games, it may be that athletes, trainers and coaches are less likely to report concussion because the athlete may be required to be pulled from the competitive event so the potential for response bias exists. Notably, 31% of all injuries from the 2010 Olympics were not reported (19). Furthermore, athletes who experience or recognize symptoms later (as is likely the case with sled head) may not have been captured in these epidemiologic studies.

We further acknowledge the limitations in our considerations of “sled head,” which is informed by clinical practice and lived experience in the world of sport and not academic evidence. Indeed, the lack of documentation within the academic and medical literature is jarring considering the extent to which the athletes are aware of the issue. As mentioned, in conducting a literature review we can only describe the current state of knowledge and recommend further investigation.

## Conclusion

Concussions and head injuries are an important, hitherto, scarely recognized concern for sledding sports athletes. Given the descriptions and concern among athletes and the lack of information in the literature, we strongly feel that investigations are warranted into the condition known as sled head. A number of interventions, if instituted, have the potential to save lives and reduce the burden of injuries in these athletes and make the sport more accessible to many more people.

## Author contributions

MM performed the review and drafted the manuscript. MC conceived of the idea, assisted in the review design and edited versions of the manuscript. Both authors approved the final manuscript.

### Conflict of interest statement

MC: Is a voluntary (unpaid) member of Parachute Canada's National Expert Advisory Committee and the National Expert Advisory Committee on Concussion and a practicing neurosurgeon. MM has no disclosures to report.
